# The Bright Fluorescent Protein mNeonGreen Facilitates Protein Expression Analysis *In Vivo*

**DOI:** 10.1534/g3.116.038133

**Published:** 2017-01-20

**Authors:** Lola Hostettler, Laura Grundy, Stéphanie Käser-Pébernard, Chantal Wicky, William R. Schafer, Dominique A. Glauser

**Affiliations:** * Department of Biology, University of Fribourg, 1700 Fribourg, Switzerland; † Neurobiology Division, MRC Laboratory of Molecular Biology, Cambridge CB2 0QH, UK

**Keywords:** nematode, worm, in vivo imaging, expression reporter, bright fluorescent protein

## Abstract

The Green Fluorescent Protein (GFP) has been tremendously useful in investigating cell architecture, protein localization, and protein function. Recent developments in transgenesis and genome editing methods now enable working with fewer transgene copies and, consequently, with physiological expression levels. However, lower signal intensity might become a limiting factor. The recently developed mNeonGreen protein is a brighter alternative to GFP *in vitro*. The goal of the present study was to determine how mNeonGreen performs *in vivo* in *Caenorhabditis elegans*—a model used extensively for fluorescence imaging in intact animals. We started with a side-by-side comparison between cytoplasmic forms of mNeonGreen and GFP expressed in the intestine, and in different neurons, of adult animals. While both proteins had similar photostability, mNeonGreen was systematically 3–5 times brighter than GFP. mNeonGreen was also used successfully to trace endogenous proteins, and label specific subcellular compartments such as the nucleus or the plasma membrane. To further demonstrate the utility of mNeonGreen, we tested transcriptional reporters for nine genes with unknown expression patterns. While mNeonGreen and GFP reporters gave overall similar expression patterns, low expression tissues were detected only with mNeonGreen. As a whole, our work establishes mNeonGreen as a brighter alternative to GFP for *in vivo* imaging in a multicellular organism. Furthermore, the present research illustrates the utility of mNeonGreen to tag proteins, mark subcellular regions, and describe new expression patterns, particularly in tissues with low expression.

The implementation of Green Fluorescent Protein (GFP), from the jellyfish *Aequorea victoria*, revolutionized cell and developmental biology research (Chudakov *et al.* 2010). Fluorescent proteins are particularly useful genetically encoded tags to visualize gene products and cellular compartments in living cells and organisms. In order to improve emitted signals and make them versatile tools, diverse fluorescent protein variants with different spectral and photophysical properties have been developed (Tsien 2009; Shaner *et al.* 2005; Shaner *et al.* 2007). Most of them derive from the *A*. *victoria* protein backbone. Recently, Shaner and collaborators have engineered the amphioxus *Branchiostoma lanceolatum* multimeric yellow fluorescence protein (LanYFP) to produce the monomeric mNeonGreen protein (Shaner *et al.* 2013). mNeonGreen is up to three times brighter than GFP *in vitro*. mNeonGreen excitation and emission peaks are slightly shifted toward higher wavelengths as compared to classical GFPs, but remain compatible with standard “GFP” filter sets used for microscopy. However, whether these promising properties will translate in better performances in living organisms remains unclear.

*C*. *elegans* was the first model organism in which GFP was expressed (Chalfie *et al.* 1994). Its amenability to genetic manipulations, its small size, and its transparent body, have made, and still make, *C*. *elegans* particularily appropriate for live cell imaging approaches in intact animals (Hobert and Loria 2006). The success of GFP imaging relies on the ability to detect relevant fluorescence signals over background signals. Tissue autofluorescence is usually the limiting parameter *in vivo*. For decades, transgenic worms carrying multiple transgene copies in extrachromosomal or genome-integrated arrays have been used, and satisfactory GFP signals recurrently obtained. Recent methodological developments, such as Mos1-mediated single-copy insertion (MosSCI) (Frokjaer-Jensen *et al.* 2008), and CRISPR-mediated genome editing (Chen *et al.* 2013; Chiu *et al.* 2013; Friedland *et al.* 2013; Frokjaer-Jensen 2013; Katic and Grosshans 2013; Tzur *et al.* 2013; Waaijers *et al.* 2013; Arribere *et al.* 2014; Kim *et al.* 2014; Zhao *et al.* 2014; Paix *et al.* 2015; Xu 2015; Dickinson and Goldstein 2016; Schwartz and Jorgensen 2016; Dickinson *et al.* 2013), enable working with fewer transgene copies. One direct benefit of these approaches is to obtain more physiological protein expression levels. However, for fluorescent proteins, this also means lower signal intensities, which can only be compensated by using brighter fluorescent proteins.

The goal of the present study was to evaluate mNeonGreen performances for *in vivo* applications in various *C*. *elegans* tissues. We found that mNeonGreen had similar photostability, but was markedly brighter than GFP *in vivo*. mNeonGreen worked very well to tag proteins, label specific subcellular compartments, and report expression patterns of low-expression genes. We conclude that mNeonGreen represents a valuable alternative to complement standard GFPs, and we have prepared a plasmid set for its dissemination in the research community.

## Materials and Methods

### Transgene construction

The design of *C*. *elegans*-tailored mNeonGreen sequence implicated codon optimization and intron integration, as previously described (Schild and Glauser 2015). The initial mNeonGreen plasmid (dg361) was obtained by gene synthesis (Genewiz). As starting plasmids for GFP constructs, we used the slot3 Gateway Entry vector pGH50 (gift from Erik Jorgensen). The 3×Flag tags were added by whole plasmid amplification with primer pairs, in which each primer contained half of the 3×Flag sequence, followed by ligation to produce dg432 and dg399. All other fluorescent protein constructions were made from these starting plasmids, with standard restriction/ligation based cloning, and/or recombination, in the three-fragment Multisite Gateway system (Invitrogen). For transcriptional reporters, the promoter definition was based on the information available in release WS220 of Wormbase (Harris *et al.* 2010). Supplemental Material, Table S1 presents a list of the oligonucleotides used in this study. Table S2 presents a list of the plasmids used in this study as well as details on how they were constructed.

### Plasmid distribution

mNeonGreen is licensed by Allele Biotechnology (San Diego), who agreed to establish a special group license scheme for the whole *C*. *elegans* community. The *C*. *elegans*-specific mNeonGreen plasmids should be requested to the corresponding author (DAG), who coordinates licensing and distribution. Table 1 presents a list of the distributed plasmids.

**Table 1:** Selected plasmids containing *C. elegans*-specific *mNeonGreen* transgenes

Plasmid Name	Description
dg353	slot2 ENTRY vector with mNeonGreen; for expression of cytoplasmic mNeonGreen
dg356	slot2 ENTRY vector with mNeonGreen::egl-13NLS; for expression of nuclear mNeonGreen
dg357	slot2 ENTRY vector with myr::mNeonGreen; for expression of mNeonGreen at the plasma membrane
dg398	slot2 ENTRY vector with mNeonGreen::3xFLAG::stop; for expression of cytoplasmic FLAG-tagged mNeonGreen
dg397	slot3 ENTRY vector with mNeonGreen::3xFLAG::stop::unc-54UTR; for expression of C-terminal fusion of FLAG-tagged mNeonGreen

### Transgenic animals

For fluorescence intensity and photostability comparisons, as well as for CMK-1 fusion subcellular localization, we created a single-copy integrant as previously described (Frokjaer-Jensen *et al.* 2012). Transgenes were all integrated at the same locus on chromosome II (*ttTi5605*). PCR was used to verify that integration took place at the expected locus. Furthermore, to rule out additional coinsertions, we verified the number of integrated copies with quantitative PCR (Figure S1A). DNA was prepared using DNAeasy Blood & Tissue Kit (Qiagen), and qPCR performed with KAPA SYBR FAST Universal qPCR Kit (Kapa Biosystems) on a Rotor Gene Q cycler. We used one pair of primers targeting the *Y41E3*.*19* gene (present in two copies in every diploid cell). Since *unc-54* UTR was present in our transgenes, we also used a pair targeting *unc-54* UTR (present in four copies in every diploid cell of single copy integrant homozygotes). Figure S1B shows the quantitative results of the verified single insertion lines retained for the study. For transcriptional reporters, mNeonGreen targeting to subcellular compartments, and behavioral rescue experiments, we created stable lines carrying extrachromosomal arrays. The coinjection marker was unc-122p::RFP (Miyabayashi *et al.* 1999). Table S3 presents a list of the strains used in this study.

### Microscopy

All image acquisitions were made in staged, first-day adult animals that were immobilized with 0.5% sodium azide.

For green fluorescence signal intensity comparisons, photostability measures, and CMK-1 fusion protein localization, we used an Axioplan 2 Zeiss epifluorescence microscope equipped with an Axiocam camera, a 40× objective (air, NA = 0.95), and a Zeiss FITC/GFP filter set #9 (BP450-490; FT510; LP515). Illumination and exposure parameters were kept constant. Animals were grown at 23°. Relevant GFP/mNeonGreen comparative measures were made in parallel, alternating both sample types during each recording sessions. Intensity quantification was made with ImageJ using integral density values, and area-normalized background subtraction. For analyses in FLP and PLM neurons, the relevant background signal was determined in a nearby head region, and subtracted in each image. For analyses in the intestine, the subtracted background reference was a region outside of the worm body. This background does not account for intestine autofluorescence. Intestine autofluorescence was estimated in nontransgenic animals (N2) and reported separately (Figure S3).

The subcellular localization of CMK-1 fusion reporters was scored, blind to genotypes and treatments, as previously described (Schild *et al.* 2014). Briefly, staged worms grown at 20° were washed off from their plates and transferred in PCR tubes (20 μl of a dense suspension containing 2–5 worms/μl). They were incubated for 1 hr in a PCR cycler. The same batch of worms was split into sets of tubes maintained in parallel either at 20° or at 28° prior to imaging. We scored CMK-1 localization in FLP cell bodies manually by comparing the intensity in the nucleus and cytoplasm. Two categories were considered: (i) cytoplasmic = nuclear, when we could not see a darker nucleus, and (ii) cytoplasmic > nuclear, when we could see a darker nucleus. Our ability to unambiguously define the expression category varied from animal to animal due to various signal intensities and the variable distance between the FLP cell body and the highly autofluorescent gut tissue. When the signal-to-noise ratio was too low for one animal, this one was excluded from the analysis. As detailed in the result section, ambiguous situations were more frequent for GFP than for mNeonGreen.

For confocal imaging, we used a Leica TCS SPE-II confocal microscope (APO 40× oil objective, NA1.15), equipped with a 488 nm wavelength diode laser and a ET525/50m emission filter. Z-stack images were acquired across whole animal thickness. Maximal intensity projections are depicted in the figures.

### Western blot analysis

Western blots were performed on first-day adult animal lysates to compare the amounts of Flag-tagged fluorescent proteins. Equal volumes of worm pellets were directly boiled 5′ at 95° in 4× sample buffer (240 mM Tris-HCl pH 6.8, 8% SDS, 40% glycerol, 1.4 M β-mercaptoethanol, bromophenol blue), broken by bead beating 5′ and reboiled 5′ at 95°. Lysates were then recovered by centrifugation. Protein concentration was estimated after absorbance reading at 280 nm. Equal amounts of proteins were loaded on SDS/PAGE and transferred to nitrocellulose membranes for western blotting analysis. We used a M2 monoclonal anti-Flag antibody (Sigma-Aldrich F3165), and a mouse monoclonal anti-actin antibody (Sigma-Aldrich A197). Secondary antibodies were anti-mouse horseradish peroxidase (HRP) conjugated (Jackson immunoresearch 115-035-003). Chemiluminescent signals were produced using SuperSignal West Femto chemiluminescent substrate (Thermo Fisher Scientific), and detected using medical X-ray films (FUJIFILM). Expression levels were quantified with ImageJ and normalized to actin levels.

### Neuron identification

In order to identify neurons expressing mNeonGreen reporters, the Imaris software was used to create 3D reconstructions, helpful for neuronal morphology visualization. When relevant, we colabeled a defined subset of sensory neurons with a Dil staining, with Dil diluted in deionized water, as previously described (Tong and Burglin 2010). We also coinjected the transgenic comarkers [*gpa-13p*::*cmk-1*::*sl2*::*mCherry*] (dg34 plasmid, known to be expressed in AWC, ADF, ASH, and phasmid neurons) or [*tax-4p*::*cmk-1*::*sl2*::*mCherry*] (dg35 plasmid, known to mark AWC, ASI, AFD, ASG, ASJ, ASK, BAG, URX, and ASE) (Lee and Ashrafi 2008).

### Behavioral assays and mutant phenotype clustering

Noxious heat avoidance was assessed with noxious heat thermogradient assays as previously described (Glauser *et al.* 2011). Quantitative analysis of locomotion behavior was performed in adults navigating on food with the Worm Tracker 2.0 platform, as previously described (Brown *et al.* 2013). For the mutant cluster analysis based on behavioral parameters, we first identified parameters that were significantly different between *F21D12.3*(*tm1010*) and wild type (N2) with Bonferroni corrected repeated Student’s *t*-tests (47 parameters). Second, focusing on these 47 parameters, we analyzed the behavioral data of *tm1010* mutants together with the data of 305 strains (Yemini *et al.* 2013). For each parameter, we computed a *z*-score that we centered to the mean of the wild-type values. Then, we performed a hierarchical clustering of the mutant strains with computed Euclidian distances between Pearson’s correlations over the 47 parameters. The GeneE software was used to create the tree and the heat map presented in Figure S5.

### Statistics

Several independent transgenic lines (numbers indicated in the text) were always assessed in parallel. Since we detected no significant difference between these lines, results are presented in aggregate. To assess statistical significance, we used Student’s *t*-test and ANOVA, followed by Bonferroni *post hoc* tests.

### Data availability

Raw data are available upon request. The authors state that all data necessary for confirming the conclusions presented in the article are represented fully within the article.

## Results

### mNeonGreen is significantly brighter than GFP in living C. elegans animals

**Figure 1:**
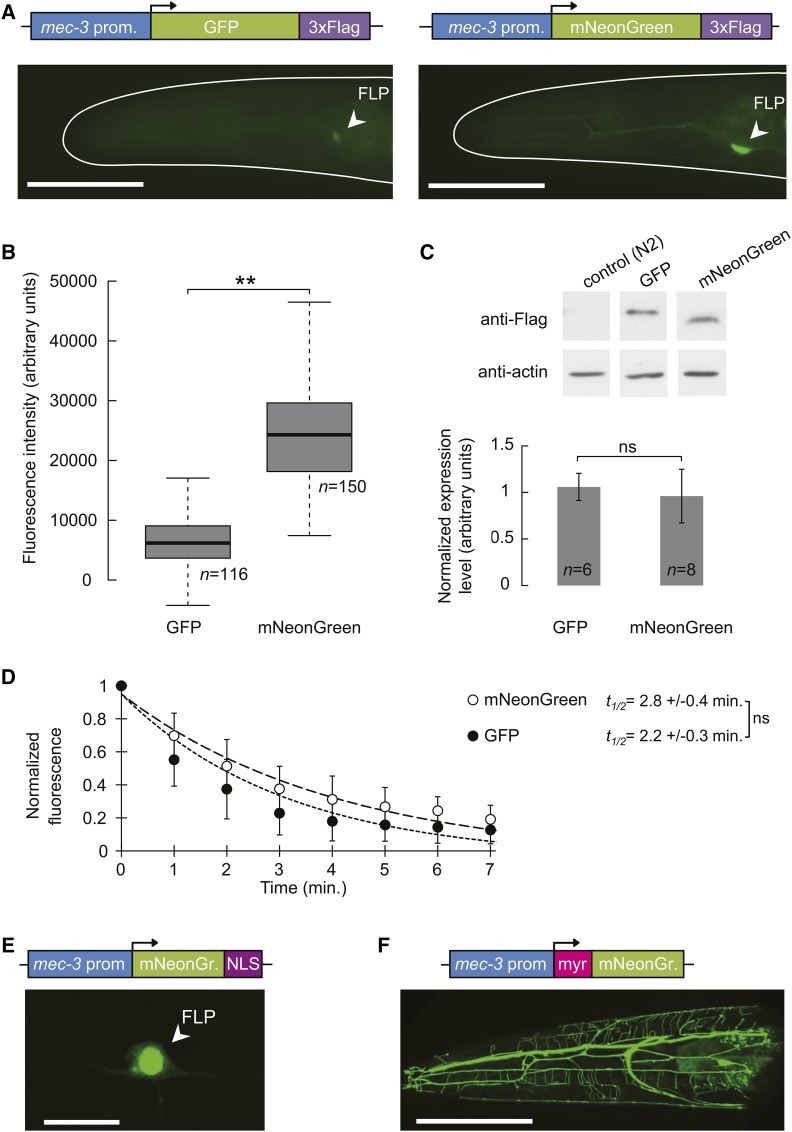
mNeonGreen is significantly brighter than GFP *in vivo*. (A) Schematics of mNeonGreen and GFP constructs, and representative epifluorescence micrographs of *C. elegans* adult heads. Bar, 50 μm. (B) Fluorescence signal quantification in FLP cell bodies. ** *P* < 0.001 by Student’s *t*-test. Neuron numbers (*n*) are indicated. (C) Representative western blot image and quantification over the indicated number (*n*) of samples. ns, not significant. The pictures were taken from the same membrane, with the same exposure conditions. (D) Photobleaching kinetics of mNeonGreen and GFP measured in FLP neuron cell bodies. Signal half-life (*t*_1/2_) was calculated for each cell body, and expressed as mean ± SEM; *n* ≥ 9. ns, not significant. (E) Schematic of the mNeonGreen nuclear targeting construct, and a representative epifluorescence micrograph showing strong nuclear expression in the nucleus of FLP neurons. Bar, 10 μm (F) Schematic of the mNeonGreen plasma membrane targeting construct, and a representative confocal micrograph projection showing the arborized neurites of the multi-dendritic FLP neuron. Bar, 50 μm.

To ensure proper expression of mNeonGreen in *C*. *elegans*, we adapted the *mNeonGreen* gene through codon optimization and integration of four artificial introns, which had also been used to enhance the expression of GFP (Fire 1995). Then, we performed a side-by-side comparison with GFP in different *C*. *elegans* tissues *in vivo*. First, we compared the fluorescence of cytoplasmic GFP and mNeonGreen expressed in specific sensory neurons. To that end, we created constructs encoding Flag-tagged versions of mNeonGreen and GFP, respectively, under the control of the *mec-3* promoter, which drives robust expression in the head nociceptive neurons FLP, and in the tail touch receptor neurons PLM (Way and Chalfie 1989). We obtained two [*mec-3p*::*GFP*::*3×Flag*] and three [*mec3-p*::*mNeonGreen*::*3×Flag*] single copy integrant worm lines, and quantified the fluorescence in neural cell bodies. mNeonGreen signal was significantly higher than GFP signal (3.5 times higher in FLP, Figure 1, A and B; and 3.2 times in PLM Figure S2). Western blot analyses showed no significant difference in expression levels between mNeonGreen and GFP (Figure 1C). Furthermore, the photobleaching kinetics of the two fluorescent proteins were not significantly different (Figure 1D).

Second, we compared the fluorescence of mNeonGreen and GFP in the intestine using the *vit-3* promoter (Heine and Blumenthal 1986). We generated four [*vit-3p*::*mNeonGreen*::*3×Flag*] and three [*vit-3p*::*GFP*::*3×Flag*] single copy transgenic lines. The signal in the GFP lines was only slightly higher than the intestinal autofluorescence detected in nontransgenic control worms (Figure S3A). In contrast, the mNeonGreen signal was almost 10 times higher than the one in the GFP lines, after subtraction of the autofluorescence (Figure S3, A and B). However, the mNeonGreen protein was also expressed at significantly higher levels (∼2 times), as shown by Western blot (Figure S3C).

Collectively, these results show that (i) *C*. *elegans*-tailored *mNeonGreen* transgene expresses well in neurons and the intestine, (ii) mNeonGreen protein photostability is similar to that of GFP *in vivo*, and (iii) at comparable expression levels, mNeonGreen produces a markedly brighter signal than GFP.

### Targeted subcellular localization of mNeonGreen

Our next goal was to evaluate if mNeonGreen could be targeted to specific subcellular compartments *in vivo*. First, we targeted mNeonGreen to the nucleus by fusing the Nuclear Localization Signal (NLS) of EGL-13 (Lyssenko *et al.* 2007) at its C-terminus. A [*mec-3p*::*mNeonGreen*::*3×Flag*::*NLS*] construct produced a fluorescent signal that was strongly enriched in the nucleus of specific sensory neurons (Figure 1E). Second, we targeted mNeonGreen to the plasma membrane by fusing a myristoylation signal at its N-terminus (Byrd *et al.* 2014). Animals carrying a [*mec-3p*::*myr*::*mNeonGreen*::*3xFlag*] transgene had a strong fluorescent signal in the plasma membrane of targeted neurons (Figure 1F). In particular, mNeonGreen very brightly labeled the complex dendritic structures of the FLP neurons (Figure 1F). These results indicate that mNeonGreen can be used to mark specific subcellular regions *in vivo*.

### mNeonGreen fusion for protein visualization

**Figure 2:**
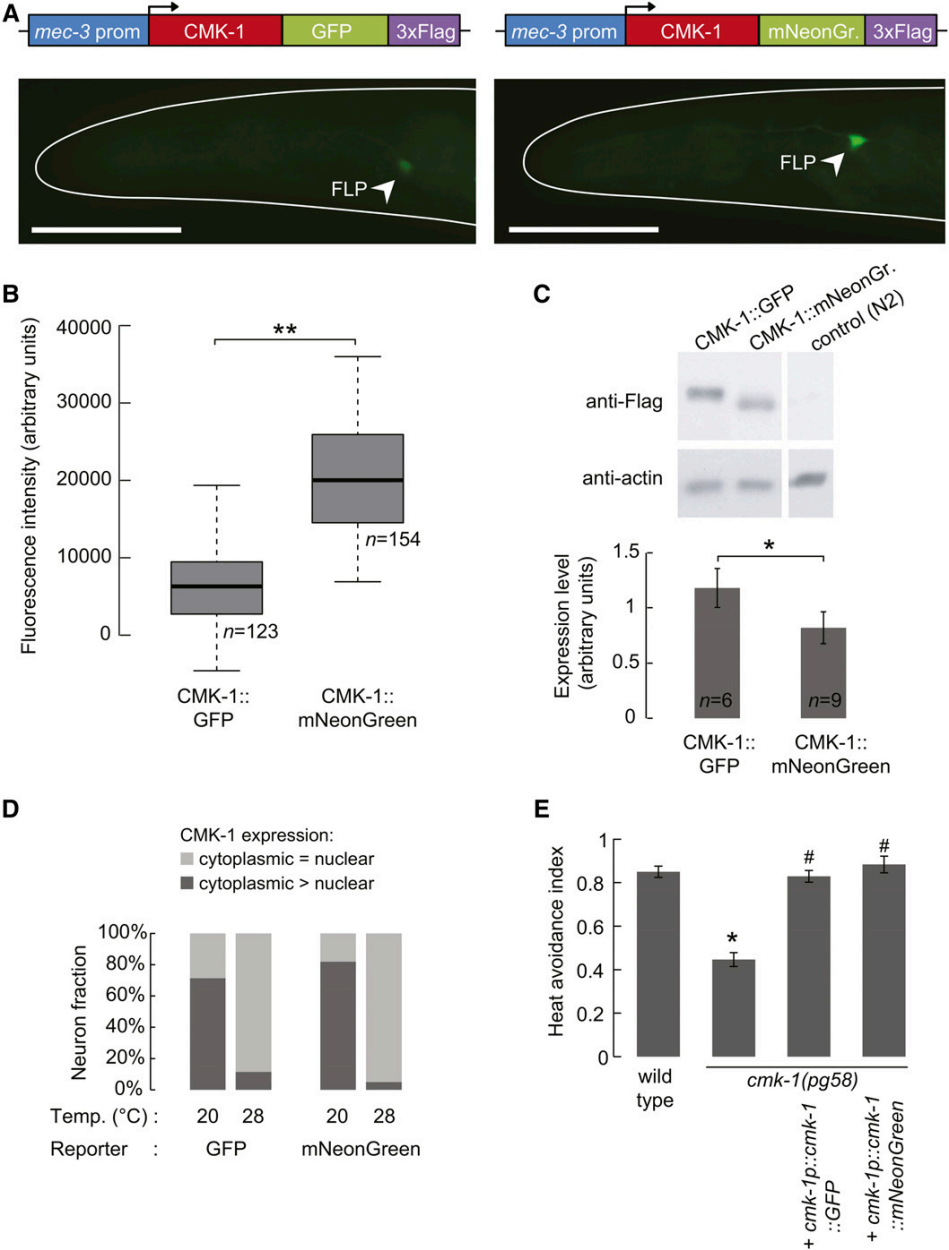
mNeonGreen tagging for protein visualization. (A) Schematics of the CMK-1 fusion construct with mNeonGreen and GFP, and representative epifluorescence micrographs of *C. elegans* adult heads. Animals were grown at 23°. Bar, 50 μm. (B) Fluorescence signal quantification in FLP cell bodies. ** *P* < 0.001 by Student’s *t*-test. Neuron numbers (*n*) are indicated. (C) Representative western blot image and quantification over the indicated number (*n*) of samples. ns, not significant. The N2 control pictures were taken from the same membrane, with the same exposure conditions. (D) Scoring of CMK-1 subcellular localization with mNeonGreen or GFP fusions in animals grown at 20° and then incubated either at 20° or at 28° for 1 hr. *n* ≥ 91 neurons. Cases for which the intensity was too low for unambiguous scoring were removed from the analysis. (E) Heat avoidance index in wild type (N2), and *cmk-1(pg58)* homozygous mutant animals. The behavioral defect is rescued by expression of CMK-1::mNeonGreen and CMK-1::GFP. Bars represent mean ± SEM. A one-way ANOVA showed significant differences across genotypes (*P* < 0.001), and was followed by Bonferroni *post hoc* tests. * *P* < 0.01 *vs.* wild type; ^#^ *P* < 0.01 *vs.* *cmk-1*(*pg58*).

Next, we wanted to evaluate the performances of mNeonGreen as a fusion tag. Previously, we had found that the Ca^2+^/Calmodulin-dependent protein kinase-1 (CMK-1) translocated to the nucleus of the FLP thermal nociceptor neurons upon prolonged (1 hr) exposure to a noxious temperature of 28° (Schild *et al.* 2014). These observations were made with a [*mec-3p*::*CMK-1*::*GFP*] construct producing relatively weak signals, such that only a subset of animals with the highest expression level could be used for unambiguous localization scoring. We wondered whether mNeonGreen could facilitate this type of experiment. Thus, we generated five [*mec-3p*::*CMK-1*::*mNeonGreen*::*3×Flag*] and four [*mec-3p*::*CMK-1*::*GFP*::*3×Flag*] single-copy integrant lines. First, we quantified the fluorescence produced by the fusion constructs. As before, the mNeonGreen signal was significantly brighter than that of GFP (∼3 times, Figure 2, A and B), even though Western blot analyses indicated a slightly (∼30%) lower expression of the mNeonGreen fusion (Figure 2C). Second, we compared the subcellular localization of GFP- and mNeonGreen-tagged CMK-1. We obtained identical results with both fluorescent tags: namely a heat-dependent relocalization of CMK-1, which went from a predominant cytoplasmic localization at 20°, to an even distribution between the cytoplasm and the nucleus at 28° (Figure 2D). Of note, with the mNeonGreen lines, the fraction of worms with insufficient signal for scoring dropped by a factor of five in comparison to the GFP lines (from 50 to 10%); this markedly reduced the time needed for scoring. Finally, we wanted to verify that the CMK-1::mNeonGreen fusion was functional. Previously, we had found that a noxious heat avoidance defect in *cmk-1*(*pg58*) mutants could be rescued by a CMK-1::GFP fusion expressed under the control of the *cmk-1* promoter (Schild *et al.* 2014). Here, we replicated this rescue experiment and quantified noxious heat avoidance with thermogradient behavioral assays. We found that, like GFP fusion, the CMK-1::mNeonGreen fusion could fully rescue the heat avoidance defect of the mutant (Figure 2E).

In summary, these data with CMK-1 fusion proteins illustrate how the strong fluorescence of mNeonGreen can facilitate protein subcellular localization studies, without impairing the function of the tagged protein.

### Uncovering unknown expression patterns using mNeonGreen reporters

**Figure 3:**
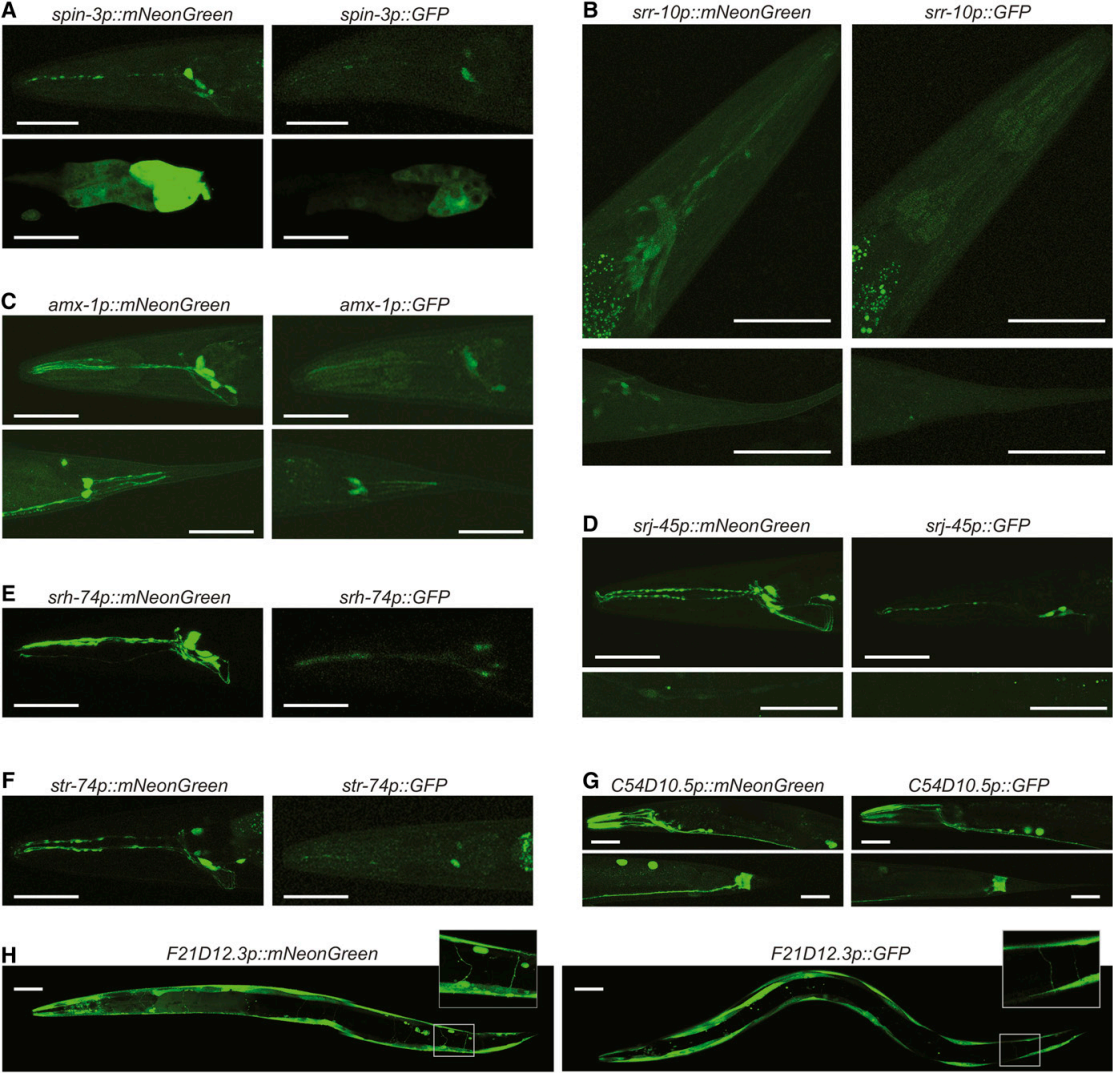
Comparison between mNeonGreen and GFP transcriptional reporters. Representative confocal projections used to determine the expression patterns in Table 2, and to compare mNeonGreen with GFP. All images were acquired with the same exposure parameters, with the exception of *str-74p*, for which *str-74*::*GFP* exposure was higher [(F), right panel]. Coelomocytes, labeled with RFP due to the coinjection marker used, produced cross-talk signal. Based on complementary epifluorescence microscopy observations, we have no evidence that any reporter is expressed in coelomocytes. Bar, 40 μm. (A, B, C, G) Head and tail close-ups in the top and bottom panels, respectively. (E, F) Head close-ups. (D) Head close-ups (top), and midbody close-ups highlighting seam cells (bottom). (H) Whole animal pictures, with insets highlighting motor neuron commissures.

Next, we evaluated if the bright signal produced by mNeonGreen would improve our ability to describe gene expression patterns. To that end, we targeted challenging candidate genes with uncharacterized expression patterns. We randomly selected 10 genes for which a previous large scale reporter screen had produced no detectable expression patterns (Hunt-Newbury *et al.* 2007), and for which no new expression pattern had been annotated in the meantime in Wormbase (Harris *et al.* 2010). We were able to clone nine out of 10 promoters and, for each, generated four mNeonGreen and four GFP transgenic lines. We detected mNeonGreen expression for all nine genes (Figure 3). Table 2 summarizes expression patterns. Across these lines, we detected fluorescence signal in a large palette of adult tissues including neurons, intestine, muscle, glands, and epithelial cells (seam cells).

**Table 2:** Expression patterns of mNeonGreen transcriptional reporters

Gene	Sequence	Expression Pattern
*F21D12.3*	F21D12.3	Body wall muscle; motor neurons; fainter expression in unidentified cells between the bulbs of the pharynx
*str-74*	C14H10.4	Six head neurons, including two amphid neuron pairs
*srh-74*	C45B11.4	Two amphid neuron pairs: ASE, AWA; two phasmid neuron pairs: PHA, PHB
*spin-3*	F09A5.1	Four most posterior cells of the gut; Head neurons: ASE, AWA, AFD and one pair of neurons in the retrovesicular ganglion
*C54D10.5*	C54D10.5	Anal depressor muscle; subset of 12 striated head muscles (eight external, four internal); neurons: two retrovesicular ganglion neurons, two neurons from the ventral ganglion, at least 16 neurons from the lateral ganglia, DVA or DVC
*srr-10*	T05B11.6	Three rectal glands; Faint expression in head and tail neurons: at least six neurons in the lumbar ganglion, at least 20 neurons in the anterior ganglion, at least 20 neurons in the lateral ganglia, two neurons in the dorsorectal ganglion
*srj-45*	T03D3.6	Neurons: AVM and six head neurons including two amphid neuron pairs; Seam cells
*angl-1*	W02G9.5	Intestine; body wall muscles; pharynx muscles; neurons: at least 12 pairs of neurons in the head lateral ganglia, five pairs of post-anal neurons, PDB, ventral nerve cord motor neurons
*amx-1*	R13G10.2	Four pairs of amphid neurons: ASJ and most likely ASH, ASE, and AWB; Phasmid neurons: PHA and PHB

Next, we compared mNeonGreen lines with GFP lines. Figure 3 shows representative confocal microscope image projections. Overall, expression patterns and signal intensities were relatively homogenous within each replicate line set. One exception was *angl-1*, for which both GFP and mNeonGreen expression levels were very variable across lines and animals (Figure S4). For the other eight genes, which could be reliably compared, GFP reporter lines overall produced dimmer signals (Figure 3). As a consequence, we observed mNeonGreen signals in tissues/cells for which GFP signal was undetectable. Most striking examples are the head neurons for *srr-10* reporters (Figure 3B), and seam cells for *srj-45* reporters (Figure 3D).

Finally, we wondered whether the mNeonGreen reporter-derived expression patterns could help determining some new gene functions. *F21D12.3* encodes a predicted transmembrane amino acid transporter related to the GABA transporter UNC-47 (McIntire *et al.* 1997). *F21D12.3 is* expressed in motor neurons and muscles (Figure 3IH and Table 2), suggesting it might control locomotion. To test this hypothesis, we used the Worm Tracker 2.0 platform (Brown *et al.* 2013) to quantify worm shape, posture, and motion in wild type and *F21D12.3*(*tm1010*) mutant animals. Out of 726 examined parameters, 47 significantly differed in mutants (*P* < 0.001 with Bonferroni correction for multiple comparisons). The most salient features of *F21D12.3*(*tm1010*) mutants included: a slightly smaller size (Figure S5A), an altered postural bending (with reduced secondary wavelength, Figure S5B), a slower locomotion (Figure S5C), and trajectory curvature biased toward the dorsal side (Figure S5D). A hierarchical clustering analysis combining these data with a database of 305 *C*. *elegans* strains (Yemini *et al.* 2013) highlighted other mutants with similar phenotype (Figure S5E). Collectively, these results illustrate that expression patterns identified with mNeonGreen reporters (Table 2) can be useful to guide the discovery of gene functions.

## Discussion

The present study provides a side-by-side comparison of GFP and mNeonGreen in *C*. *elegans*. Across different adult worm tissues, and different protein contexts (free cytoplasmic version or protein fusion), mNeonGreen was markedly brighter than GFP. Apart from its increased brightness, mNeonGreen behaved like GFP in all the other examined aspects. Indeed, mNeonGreen had similar photostability, expressed well across many tissues, could be targeted to specific subcellular regions, and could be used as a tag to visualize changes in protein subcellular localization *in vivo*. With the growing application of efficient single-copy transgene integration methods (Frokjaer-Jensen *et al.* 2008, 2012) and the boom in genome editing techniques that enable the addition of fluorescent tags to endogenous coding sequences (Dickinson *et al.* 2013; Tzur *et al.* 2013; Kim *et al.* 2014; Paix *et al.* 2014), the development of brighter fluorescent tags will fulfill an important emerging need. Stronger signal can ease expression pattern definition, as illustrated with our reporter approach in low-expression genes, and simplify protein tracking within cells *in vivo*, as we illustrated with CMK-1 experiments. Furthermore, we anticipate it could improve the visualization of small cellular structures, and facilitate high throughput experiments, where time is constraining, or when fluorescence detection sensitivity is limiting.

Because mNeonGreen and GFP have slightly different spectral properties, the apparent brightness increase with mNeonGreen will depend on the optical parameters of the imaging setup used. The quantification reported here was made with a microscope equipped with a very common “FITC/GFP” filter set (see *Materials and Methods*). While we have not made any quantitative comparisons with other platforms, the increased brightness of mNeonGreen was also obvious with a Leica TCS SPE-II confocal microscope and a Leica dissecting scope (with either GFP3 or GFP2 filter sets). We anticipate that the mNeonGreen benefits could be readily accessible to most, if not all, laboratories presently imaging GFP.

While protein expression was similar in neurons, we observed stronger expression levels for mNeonGreen as compared to GFP in the intestine, despite using transgenes integrated at the same location in the genome, and that differed only in their fluorescent protein coding sequences. We do not know if this tissue-specific effect is due to increased mRNA expression, protein synthesis, or protein stability. In a recent report, Heppert and collaborators compared GFP with another mNeonGreen transgene version in *C*. *elegans* (Heppert *et al.* 2016). Using different fusion constructs expressed in embryo, they recorded variable *in vivo* fluorescence level ratios between mNeonGreen and GFP, including situations where mNeonGreen was not better than GFP. Expression level ratios were also variable, but it is hard to conclude if this caused fluorescence intensity differences, since both parameters were determined at different developmental stages in this study (Heppert *et al.* 2016). Taken together, the results of Heppert *et al.* 2016 and of the present study suggest that mNeonGreen and GFP can both be affected by tissue-specific factors influencing expression and/or resulting signals. While mNeonGreen might be equivalent to GFP for strong expression levels, it represents a valuable alternative, for example, in a number of nonembryonic tissues examined in the present study.

Here, we challenged mNeonGreen with a difficult set of reporter genes, which had previously failed to work with GFP in a large-scale survey (Hunt-Newbury *et al.* 2007). Because the expression was quite strong in most strains and tissues, including with GFP, our success can most probably be imputed to the redefinition of better promoter sequences as compared to the previous large-scale screen. However, parts of identified patterns were detectable with mNeonGreen, and were missed with GFP. Furthermore, the bright mNeonGreen signal greatly eased the identification of the cells and tissues composing the different expression patterns. We anticipate that mNeonGreen could help uncover detailed expression patterns.

In conclusion, we demonstrated that mNeonGreen is a brighter alternative to GFP in several *C*. *elegans* adult tissues, and have so far identified no disadvantage for its use *in vivo*. We have created several general purpose plasmids (Table 1) that we are glad to distribute to the community upon request (see *Materials and Methods*). We hope that this will facilitate the implementation of this tool to complement other available GFPs.

## Supplementary Material

Supplemental material is available online at www.g3journal.org/lookup/suppl/doi:10.1534/g3.116.038133/-/DC1
