# Supplementary material for: The Bright Fluorescent Protein mNeonGreen Facilitates Protein Expression Analysis In Vivo
Source: G3 (Bethesda). 2017 Jan 20;7(2):607–15. doi: 10.1534/g3.116.038133 (PMC5295605; doi:10.1534/g3.116.038133)
Supplement: Supplementary file 1 [file 607FigS1.pdf]

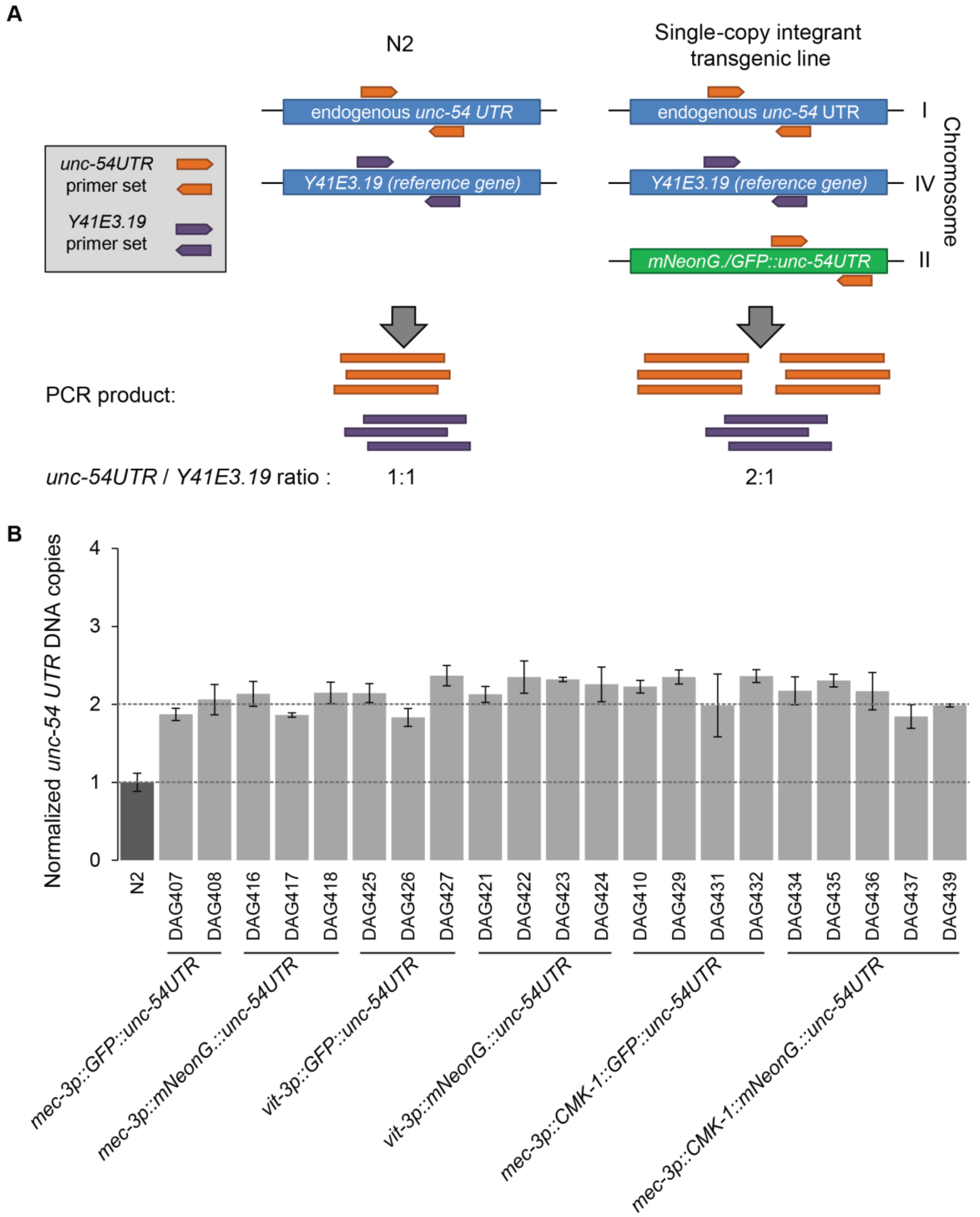

Fig. S1 Assessment of the transgene copy number in single-copy integrant lines. (A) qPCR strategy for assessing the number of transgene copies that were integrated. Two primer sets are used in parallel reactions. One primer set (purple) amplifies a control region in the reference gene *Y41E3.19*. A second primer set (orange) amplifies a genomic region in *unc-54* UTR. Since *unc-54* UTR is included in both mNeonGreen and GFP constructs, an extra copy of this sequence is present in single-copy integrant transgenic animals. (B) qPCR results expressed as averages relative copy numbers (+/- s.e.m), normalized to the *Y41E3.19* reference gene (*n*=3 replicates). In non-transgenic animals (N2), the *unc-54* UTR/*Y41E3.19* ratio was 1; in single-copy integrants, this ratio was close to the theoretical value of 2.
