# Supplementary material for: The Bright Fluorescent Protein mNeonGreen Facilitates Protein Expression Analysis In Vivo
Source: G3 (Bethesda). 2017 Jan 20;7(2):607–15. doi: 10.1534/g3.116.038133 (PMC5295605; doi:10.1534/g3.116.038133)
Supplement: Supplementary file 2 [file 607FigS2.pdf]

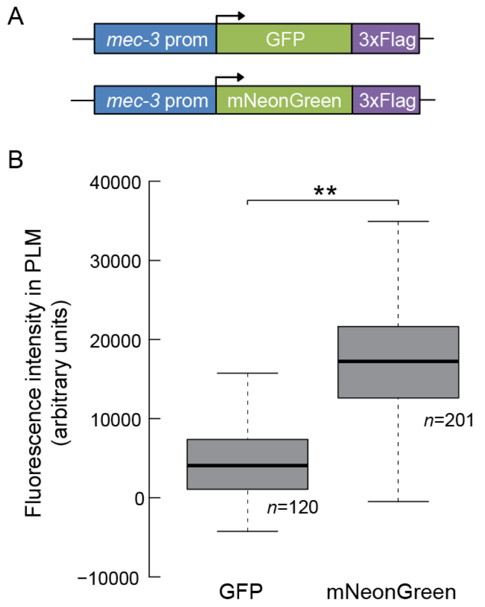

Fig. S2 mNeonGreen is significantly brighter than GFP *in vivo* in the PLM neurons of *C. elegans*. (A) Schematics of mNeonGreen and GFP constructs. (B) Fluorescence signal quantification in PLM cell bodies. **, $p<.001$ by Student's *t*-test. Neuron numbers (*n*) are indicated.
