# Supplementary material for: The Bright Fluorescent Protein mNeonGreen Facilitates Protein Expression Analysis In Vivo
Source: G3 (Bethesda). 2017 Jan 20;7(2):607–15. doi: 10.1534/g3.116.038133 (PMC5295605; doi:10.1534/g3.116.038133)
Supplement: Supplementary file 3 [file 607FigS3.pdf]

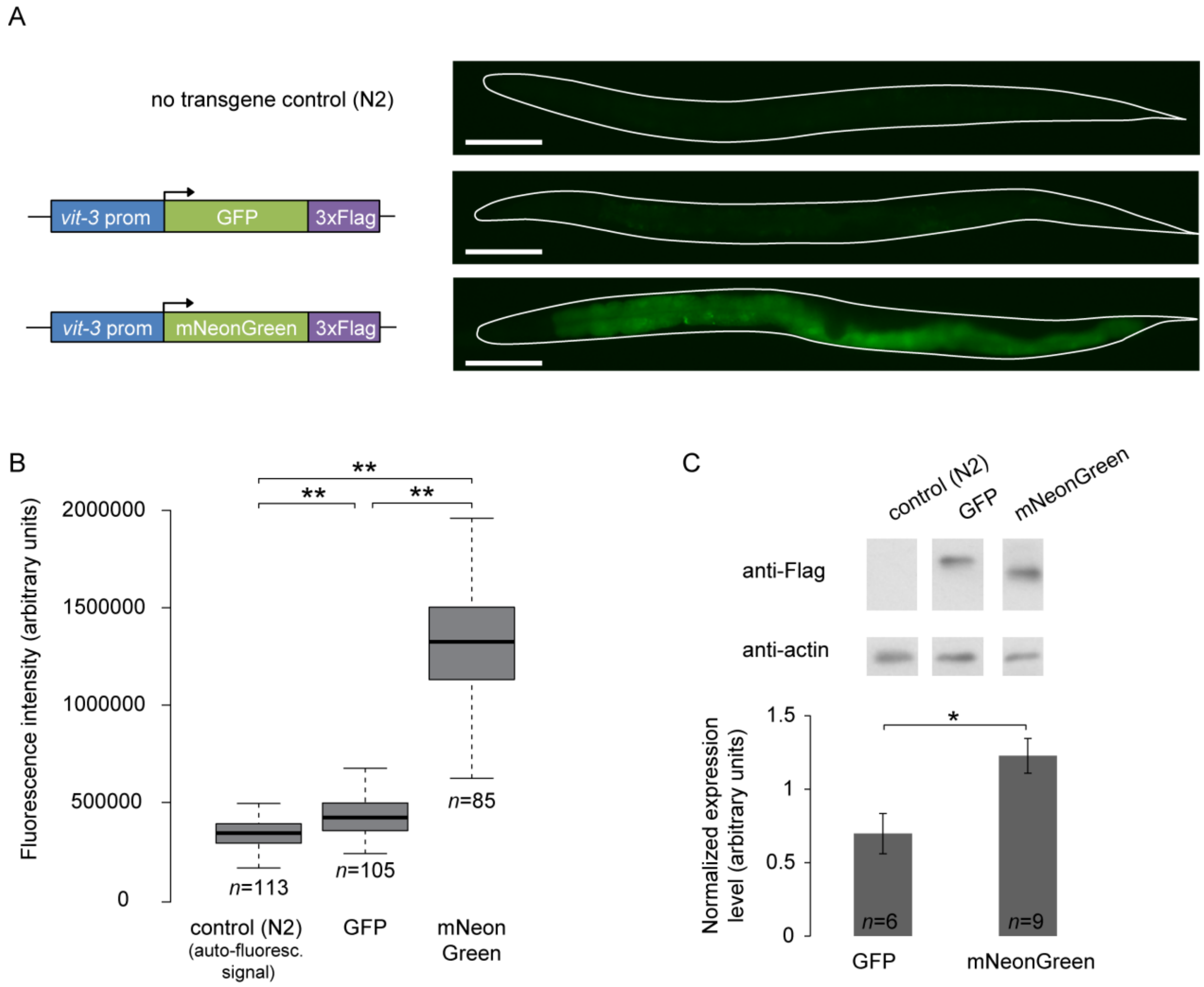

Fig. S3 mNeonGreen is significantly brighter than GFP *in vivo* in the intestine of *C. elegans*. (A) Schematics of mNeonGreen and GFP constructs and representative confocal projections of *C. elegans* adult animals. Scale bar: 100 µm. The animal contour was drawn (white line). (B) Fluorescence signal quantification in the intestine. Significant effect of the genotype by one-way ANOVA; **, p<.001 by Bonferroni post hoc tests. Animal numbers (n) are indicated. (C) Representative western blot images and quantifications over the indicated number (n) of samples. *, p<.01 by Student's t-test. The pictures were taken from the same membrane, with the same exposure conditions.
