# Supplementary material for: The Bright Fluorescent Protein mNeonGreen Facilitates Protein Expression Analysis In Vivo
Source: G3 (Bethesda). 2017 Jan 20;7(2):607–15. doi: 10.1534/g3.116.038133 (PMC5295605; doi:10.1534/g3.116.038133)
Supplement: Supplementary file 4 [file 607FigS4.pdf]

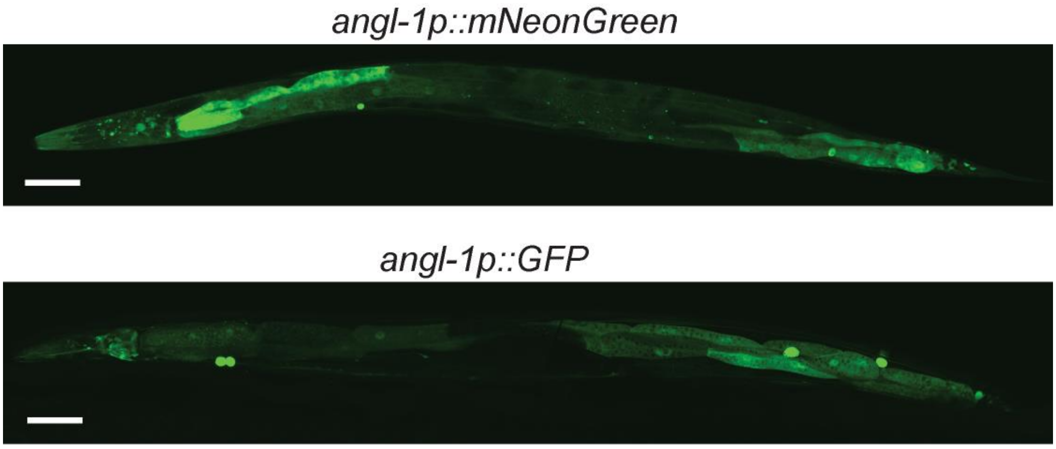

Fig. S4 *angl-1* mNeonGreen and GFP transcriptional reporters. Confocal projections of mNeonGreen and GFP reporters for *angl-1*. Expression levels were very variable and mosaicism particularly high for this set of reporter lines. These pictures were selected as the most comprehensive in term of tissue labelling.
