# Supplementary material for: The Bright Fluorescent Protein mNeonGreen Facilitates Protein Expression Analysis In Vivo
Source: G3 (Bethesda). 2017 Jan 20;7(2):607–15. doi: 10.1534/g3.116.038133 (PMC5295605; doi:10.1534/g3.116.038133)
Supplement: Supplementary file 5 [file 607FigS5.pdf]

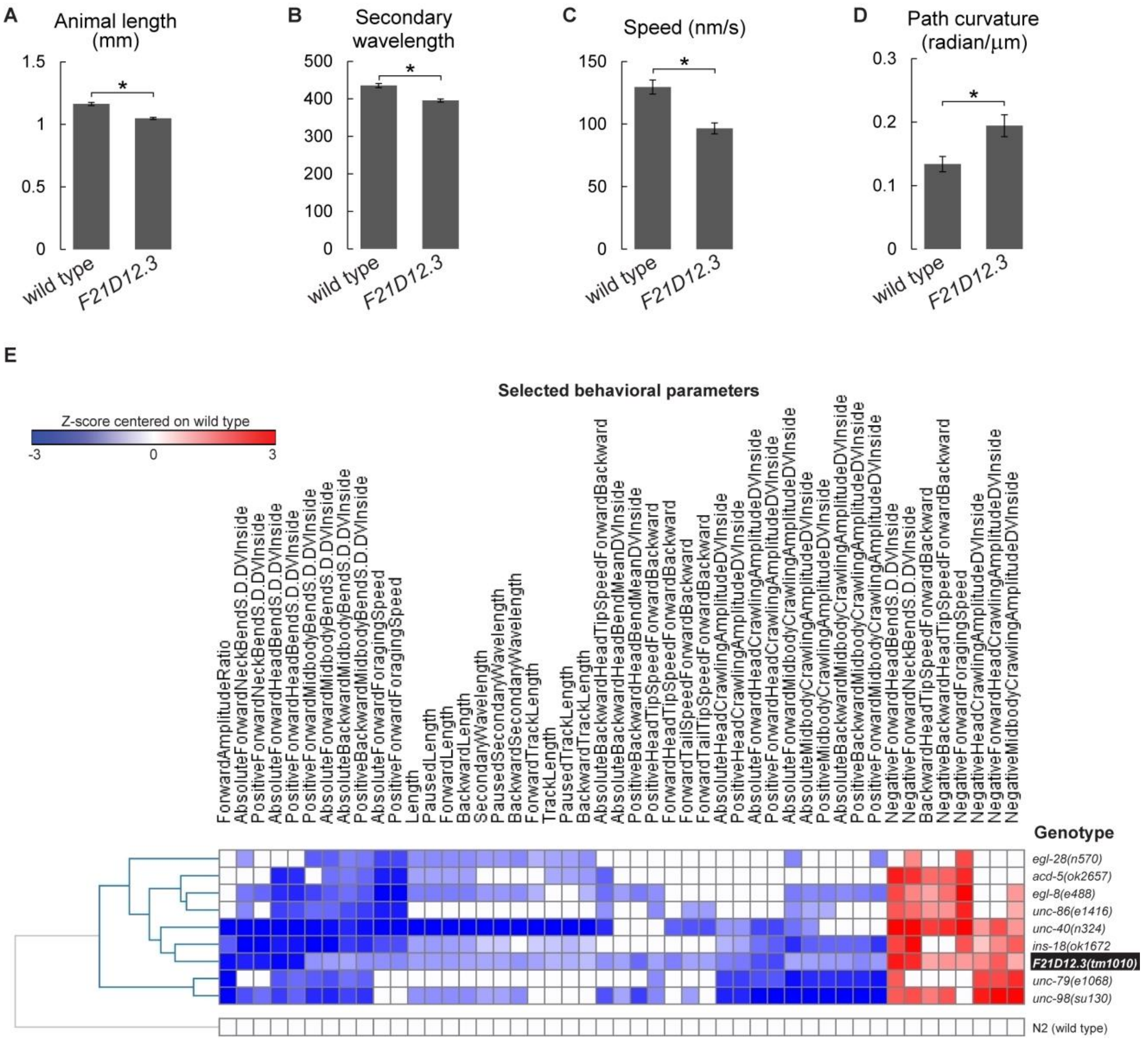

Fig. S5 ***F21D12.3*** **controls locomotion** (A, B, C, D) Selected posture and locomotion parameters affected by the *F21D12.3(tm1010)* mutation, as compared to wild type (N2). Data were derived from behavioral movies. *n*=28 animals per strain. Secondary wavelength refers to the wavelength associated with the second-largest body bend (Cronin et al. 2005). The path curvature is defined as the average angle of bearing of worm's path divided by the distance traveled. The curvature is signed relative to -ventral-dorsal orientation: when the worm's path curves in the direction of its ventral side, the curvature is signed negatively (Yemini et al., 2013). Thus, the *F21D12.3* mutant has a more pronounced bias to curve toward the dorsal side than wild type. (E) Heat map reflecting the behavioral phenotypic pattern of *F21D12.3* mutants and of the eight most similar mutants among a 305 strain database. Only behavioral parameters showing significant differences between *F21D12.3* and wild type are depicted.
