# Supplementary material for: The Bright Fluorescent Protein mNeonGreen Facilitates Protein Expression Analysis In Vivo
Source: G3 (Bethesda). 2017 Jan 20;7(2):607–15. doi: 10.1534/g3.116.038133 (PMC5295605; doi:10.1534/g3.116.038133)
Supplement: Supplementary file 6 [file 607TableS1.docx]

**TABLE S1: Oligonucleotides used in this study**

Part 1 (cloning oligos)

| Name of oligo | Sequence | Used for |
| --- | --- | --- |
| *NGflag_F* | catgacatcgattacaaggatgacgatgacaagactagtatgagccgtagacgaaaagc | 3xFlag addition to the mNeonGreen starting plasmid dg361. Plasmid created: dg432 |
| *NGflag_R* | atctttataatcaccgtcatggtctttgtagtccttgtagagttcatccattcccatcaca |  |
| *attb1NG_F* | ggggacaagtttgtacaaaaaagcaggcttaatggtgtcgaagggagaagag | Cloning of Slot2 ENTRY vector for mNeonGreen::3xFlag  Plasmid created: dg398 |
| *attb2flag_R* | ggggaccactttgtacaagaaagctgggtcttacttgtcatcgtcatccttgtaatcgat |  |
| *GFPflag_F* | catgacatcgattacaaggatgacgatgacaagactagttagcattcgtagaattccaactga | Cloning of Slot3 ENTRY vector for GFP::3xFlag  Plasmid created: dg399 |
| *GFPflag_R* | atctttataatcaccgtcatggtctttgtagtctttgtatagttcatccatgccatgtgtaatc |  |
| *attb1GFP_F* | ggggacaagtttgtacaaaaaagcaggcttaatgagtaaaggagaagaacttttcactggagtt | Cloning of Slot2 ENTRY vector for GFP::3xFlag  Plasmid created: dg400 |
| *attb2flag_R* | ggggaccactttgtacaagaaagctgggtcttacttgtcatcgtcatccttgtaatcgat |  |
| *srh-74p_F* | ggggacaactttgtatagaaaagttgttcacgaacagacattgccaatgaag | Cloning of Slot1 ENTRY vector for *srh-74p*  Plasmid created: dg407 |
| *srh-74p_R* | ggggactgcttttttgtacaaacttgtaaaaacaccctggctatgttggg |  |
| *spin3p_F* | ggggacaactttgtatagaaaagttggaacagaaacctgctagaatgtgagg | Cloning of Slot1 ENTRY vector for *spin-3p*  Plasmid created: dg408 |
| *spin-3p_R* | ggggactgcttttttgtacaaacttgatttgaccctgaaagtgttcaacagt |  |
| *C54D10.5p_F* | ggggacaactttgtatagaaaagttgtaggttttaagggtctatataccaacatt | Cloning of Slot1 ENTRY vector for *C54D10.5p*  Plasmid created: dg409 |
| *C54D10.5p_R* | ggggactgcttttttgtacaaacttgtgattgttggatctaaaatgaacactattg |  |
| *srr-10p_F* | ggggacaactttgtatagaaaagttggcagataaaacaagtgatttatgagacag | Cloning of Slot1 ENTRY vector for *srr-10p*  Plasmid created: dg410 |
| *srr-10p_R* | ggggactgcttttttgtacaaacttgtccaaaacttttaaaatcaagtcaactcc |  |
| *srj-45p_F* | ggggacaactttgtatagaaaagttgagtcatattatccatccaaccgttgtatt | Cloning of Slot1 ENTRY vector for *srj-45p*  Plasmid created: dg411 |
| *srj-45p_R* | ggggactgcttttttgtacaaacttgcgtaatttcagcacaggttttgtctt |  |
| *angl-1p_F* | ggggacaactttgtatagaaaagttgacgaattcccgcgaattctggagtt | Cloning of Slot1 ENTRY vector for *angl-1p*  Plasmid created: dg412 |
| *angl-1p_R* | ggggactgcttttttgtacaaacttgcgtcctgccacgtcgaggatatt |  |
| *F21D12.3p_F* | ggggacaactttgtatagaaaagttgagatattcctcccacctactataagc | Cloning of Slot1 ENTRY vector for *F21D12.3p*  Plasmid created: dg413 |
| *F21D12.3p_F* | ggggactgcttttttgtacaaacttgtatgataactcctgtaagattgggtaagaa |  |
| *amx-1p_F* | ggggacaactttgtatagaaaagttgtcaggtcgcctaggtaacaagca | Cloning of Slot1 ENTRY vector for *amx-1p*  Plasmid created: dg414 |
| *amx-1p_R* | ggggactgcttttttgtacaaacttgtgcacaggcgcgttttacg |  |
| *str-74p_R* | ggggacaactttgtatagaaaagttgctcctcctcatcccacatc | Cloning of Slot1 ENTRY vector for *str-74p*  Plasmid created: dg415 |
| *str-74p _F* | ggggactgcttttttgtacaaacttgtctcgatatacagacttttcaagtaaaaca |  |

**TABLE S1: Oligonucleotides used in this study** (continued)

Part 2 (qPCR and sequencing oligos)

| Name of oligo | Sequence | Used for |
| --- | --- | --- |
| *Forward:*  *oSKP-144* | gacgaaaagtcaaattttccaa | qPCR amplification of *Y41E3.19* reference gene (present as one endogenous copy in each haploid genome) |
| *Reverse:*  *oSKP-145* | tgggggatcaagtcaaggta |  |
| *Forward:*  *unc-45UTRF* | tcacaagtattgatgagcacga | qPCR amplification of *unc-54* UTR sequence (present as one endogenous copy in each haploid genome, plus one extra copy in case of successful single copy transgene integration) |
| *Reverse:*  *unc-45UTRR* | actcaccttccactgagcct |  |
| *seqUTR* | ttgacaccagacaagttggt | Sequencing of unc-54UTR containing plasmids |
| *Ng306_F* | ggattccaccaatacctcccata | Sequencing of mNeonGreen coding sequence |
| *Ng714_R* | acgaccacttgaatgtcgagatga | Sequencing of mNeonGreen coding sequence |
| *cmk-1, 274_R* | gcttctcgtcgtatgtatcg | Sequencing of CMK-1 coding sequence |
| *cmk-1, 961_F* | tccgtctctcctcaaatagc | Sequencing of CMK-1 coding sequence |
| *gfp_RI* | gttttccgtatgttgcatcac | Sequencing of sequence upstream of GFP coding sequence |
