# Supplementary material for: The Bright Fluorescent Protein mNeonGreen Facilitates Protein Expression Analysis In Vivo
Source: G3 (Bethesda). 2017 Jan 20;7(2):607–15. doi: 10.1534/g3.116.038133 (PMC5295605; doi:10.1534/g3.116.038133)
Supplement: Supplementary file 7 [file 607TableS2.docx]

**TABLE S2: Plasmids used in this study**

| Plasmid name | Insert | Comment |
| --- | --- | --- |
| dg9 / coel::RFP | *unc122p::RFP* | coel::RFP was a gift from Piali Sengupta (Addgene plasmid # 8938) |
| dg34 | *gpa-13p::CMK-1::SL2::mCherry* | See Schild *et al.* 2014 |
| dg35 | *tax-4 ::CMK-1::SL2::mCherry* | See Schild *et al.* 2014 |
| dg68 | *mec-3p* | Slot1 ENTRY vector containing *mec-3p* promoter. See Schild *et al.* 2014 |
| dg286 | *cmk-1 coding sequence* | Slot2 ENTRY vector with *cmk-1* coding sequence (with start codon, without stop codon). See Schild *et al.* 2014. |
| dg353 | *mNeonGreen* | Slot2 ENTRY vector with mNeonGreen; for expression of cytoplasmic mNeonGreen. Contains start and stop codons. |
| dg356 | *mNeonGreen::NLS* | Slot2 ENTRY vector with mNeonGreen::egl-13NLS; for expression of nuclear mNeonGreen. Contains start and stop codons. |
| dg357 | *myr::mNeonGreen* | Slot2 ENTRY vector with myr::mNeonGreen; for expression of mNeonGreen to the plasma membrane. Contains start and stop codons. |
| dg361 | *myr::mNeonGreen::NLS* | Plasmid generated by gene synthesis, containing myristoylation signal sequence (*myr*) in 5’ and EGL-13 Nulcear Localization Sequence (*NLS*) in 3’. This is a starting plasmid for cloning purpose only. |
| dg397 | *mNeonGreen::3xFlag::unc-54UTR* | Slot3 ENTRY vector for *mNeonGreen::3xFlag::unc-54UTR*. Used to create C- term fusions. Contains a stop codon. |
| dg398 | *mNeonGreen::3xFlag* | Slot2 ENTRY vector for *mNeonGreen::3xFlag*. Used to create expression constructs for cytoplasmic expression of mNeonGreen. Contains start and stop codons. |
| dg399 | *GFP::3xFlag::unc-54UTR* | Slot3 ENTRY vector for *GFP::3xFlag::unc-54UTR*. Used to create C- term fusions. Contains a stop codon. |
| dg400 | *GFP::3xFlag* | Slot2 ENTRY vector for *GFP3::xFlag*. Used to create expression constructs for cytoplasmic expression of GFP. Contains start and stop codons. |
| dg401 | *mec-3p::mNeonGreen::3xFlag::unc-54UTR* | Construct for mNeonGreen expression in FLP and PLM, generated by LR reaction between dg68, dg398, pMH473, and pCFJ150. |
| dg402 | *mec-3p::GFP::3xFlag::unc-54UTR* | Construct for GFP::3xFlag expression in FLP and PLM, generated by LR reaction between dg68, dg399, pMH473, and pCFJ150 |
| dg403 | *vit-3p::mNeonGreen::3xFlag::unc-54UTR* | Construct for mNeonGreen::3xFlag expression in the intestine, generated by LR reaction between pms7, dg398, pMH473, and pCFJ150. |
| dg404 | *vit-3p::GFP::3xFlag::unc-54UTR* | Construct for GFP expression in the intestine, generated by LR reaction between pms7, dg399, pMH473, and pCFJ150. |
| dg405 | *mec-3p::cmk-1::mNeonGreen::3xFlag::unc-54UTR* | Construct for CMK-1::mNeonGreen::3xFlag expression in FLP, generated by LR reaction between dg68, dg398, pMH473, and pCFJ150. |
| dg406 | *mec-3p::cmk-1::GFP::3xFlag::unc-54UTR* | Construct for CMK-1::GFP::3xFlag expression in FLP, generated by LR reaction between dg68, dg399, pMH473, and pCFJ150. |
| dg407 | *srh-74 promoter* | Slot1 ENTRY vector for *srh-74p*. A 1.7 kb fragment was cloned by PCR followed by BP recombination into mg169 |
| dg408 | *spin-3 promoter* | Slot1 ENTRY vector for *spin-3p*. A 2 kb fragment was cloned by PCR followed by BP recombination into mg169 |
| dg409 | *C54D10.5 promoter* | Slot1 ENTRY vector for *C54D10.5p*. A 2.5 kb fragment was cloned by PCR followed by BP recombination into mg169 |
| dg410 | *srr10 promoter* | Slot1 ENTRY vector for *srr10p*. A 662 bp fragment was cloned by PCR followed by BP recombination into mg169 |
| dg411 | *srj-45 promoter* | Slot1 ENTRY vector for *srj-45p*. A 1 kb fragment was cloned by PCR followed by BP recombination into mg169 |
| dg412 | *W02G9.5 promoter* | Slot1 ENTRY vector for *W02G9.5p*. A 1.9 kb fragment was cloned by PCR followed by BP recombination into mg169 |
| dg413 | *F21D12.3 promoter* | Slot1 ENTRY vector for *F21D12.3p*. A 2 kb fragment was cloned by PCR followed by BP recombination into mg169 |
| dg414 | *amx-1 promoter* | Slot1 ENTRY vector for *amx-1p*. A 2 kb fragment was cloned by PCR followed by BP recombination into mg169 |
| dg415 | *str-74 promoter* | Slot1 ENTRY vector for *str-74p*. A 1.5 kb fragment was cloned by PCR followed by BP recombination into mg169 |
| dg416 | *srh-74p::mNeonGreen::3xFlag::unc-54UTR* | mNeonGreen transcriptional reporter generated by LR reaction between dg407, dg398, pMH473, and pCFJ150 |
| dg417 | *spin-3p::mNeonGreen::3xFlag::unc-54UTR* | mNeonGreen transcriptional reporter generated by LR reaction between dg408, dg398, pMH473, and pCFJ150 |
| dg418 | *C54D10.5p::mNeonGreen::3xFlag::unc-54UTR* | mNeonGreen transcriptional reporter generated by LR reaction between dg409 dg398, pMH473, and pCFJ150 |
| dg419 | *srr-10p::mNeonGreen::3xFlag::unc-54UTR* | mNeonGreen transcriptional reporter generated by LR reaction between dg410, dg398, pMH473, and pCFJ150 |
| dg420 | *srj-45p::mNeonGreen::3xFlag::unc-54UTR* | mNeonGreen transcriptional reporter generated by LR reaction between dg411, dg398, pMH473, and pCFJ150 |
| dg421 | *angl-1p::mNeonGreen::3xFlag::unc-54UTR* | mNeonGreen transcriptional reporter generated by LR reaction between dg412, dg398, pMH473, and pCFJ150 |
| dg422 | *amx-1p::mNeonGreen::3xFlag::unc-54UTR* | mNeonGreen transcriptional reporter generated by LR reaction between dg414, dg398, pMH473, and mg207 |
| dg423 | *F21D12.3p::mNeonGreen::3xFlag::unc-54UTR* | mNeonGreen transcriptional reporter generated by LR reaction between dg413, dg398, pMH473, and pCFJ150 |
| dg424 | *str-74p::mNeonGreen::3xFlag::unc-54UTR* | mNeonGreen transcriptional reporter generated by LR reaction between dg415, dg398, pMH473, and pCFJ150 |
| dg425 | *cmk-1p::cmk-1::mNeonGreen::3xFlag::unc-54UTR* | Construct for CMK-1::mNeonGreen fusion expression in *cmk-1* expressing cells, generated by LR reaction between mg268, dg286, dg397, and mg207 |
| dg432 | *myr::mNeonGreen::3xFlag::NLS* | 3xFlag was added to dg361 by whole plasmid PCR amplification. This plasmid is for cloning purpose only. |
| dg541 | *srr-10p::GFP::3xFlag::unc-54UTR* | GFP transcriptional reporter generated by LR reaction between dg410, dg400, pMH473, and pCFJ150 |
| dg542 | *srj-45p::GFP::3xFlag::unc-54UTR* | GFP transcriptional reporter generated by LR reaction between dg411, dg400, pMH473, and pCFJ150 |
| dg543 | *F21D12.3p::GFP::3xFlag::unc-54UTR* | GFP transcriptional reporter generated by LR reaction between dg413, dg400, pMH473, and pCFJ150 |
| dg544 | *amx-1p::GFP::3xFlag::unc-54UTR* | GFP transcriptional reporter generated by LR reaction between dg414, dg400, pMH473, and mg207 |
| dg545 | *str-74p::GFP::3xFlag::unc-54UTR* | GFP transcriptional reporter generated by LR reaction between dg415, dg400, pMH473, and pCFJ150 |
| dg546 | *C54D10.5p::GFP::3xFlag::unc-54UTR* | GFP transcriptional reporter generated by LR reaction between dg409, dg400, pMH473, and pCFJ150 |
| dg547 | *angl-1p::GFP::3xFlag::unc-54UTR* | GFP transcriptional reporter generated by LR reaction between dg412, dg400, pMH473, and pCFJ150 |
| dg548 | *srh-74p::GFP::3xFlag::unc-54UTR* | GFP transcriptional reporter generated by LR reaction between dg407, dg400, pMH473, and pCFJ150 |
| dg549 | *spin-3p::GFP::3xFlag::unc-54UTR* | GFP transcriptional reporter generated by LR reaction between dg408, dg400, pMH473, and pCFJ150 |
| mg169 | pDONR P4-P1R | Slot1 DONOR vector. Gift from Miriam Goodman. |
| mg205 | pDONR 221 | Slot2 DONOR vector. Gift from Miriam Goodman. |
| mg207 | pDEST R4-R3 | Destination vector. Gift from Miriam Goodman. |
| mg268 | *cmk-1p* | Slot1 ENTRY vector containing *cmk-1* promoter. See Schild *et al.* 2014 |
| pCFJ150 | pDESTttTi5605[R4-R3] | Destination vector for MosSCI. Gift from Erik Jorgensen (Addgene plasmid # 19329). |
| pMH473 | *GFP::unc-54UTR* | Slot3 ENTRY clone containing GFP::unc-54 UTR. Gift from Marc Hammarlund. |
